# Supplementary material for: Tandemly repeated NBPF HOR copies (Olduvai triplets): Possible impact on human brain evolution
Source: Life Sci Alliance. 2022 Oct 19;6(1):e202101306. doi: 10.26508/lsa.202101306 (PMC9584774; doi:10.26508/lsa.202101306)
Supplement: Supplementary file 1 [file LSA-2021-01306_TableS1.docx]

**Supplementary Table 1.** Human consensus NBPF monomers m1, m2 and m3 for NCBI genome assembly NC_000001.11.

human m1 NBPF consensus

CCTGAAGGCTGGTCATGATAGAAATTCCTCGGTTTTTCTCCCAGAAACTGTGGGTAAAATGTCCCTATTCTAGTAGATCGTTATCCCAATATCATTTGTCCCAAGTTTGTGCAAACAGTTATGCCATATTTTTCCAATCAACTTAAAGCAAATACCCTCAAATGATTTCTAGGAGAAAAACTGCAATATTTAGCCCTGTCTCATCAAATACTCAGATTGTTCATGGTTGTGAGGACTTTAGACACTGAAATTAGAGTGAAAAAGGAAATCTACAAACCCTTGAGTCAAAATCATAGTTCTCTGAATTTGTCACATCTGCCCAGGTCCAATGTCATGAGAATAGGATCAGGGCGCCACAGGTATGGCCTGAGACTAGGAAGAGAGTCTTGCTCACTGACCCATCCCTTGTCTGGGCTTCCAGGTAGAACTAGAGTTTCATTCAACCTACATGTGCCTATAGGTCCTCCCTGTGGCAATGACATCTCTCAGCTCAGTAATGGCCACTTGGAGCAGGAATATGATCTTTATATGGAAGACTCAGTGGATCCTTATCACCTTCATAGAAAGGTACTCACCTCCCACGTCAAGAGAAAAGCCAACATGTTTTTCCTCCAATGCATAAAAGGAACTTCCATAGGGCTGGCAGGAGTCAGGCTGTTCAAGACAACTGGAAGGAGTTGAATAACATCTATCCAGTGAGTCCTGCAAGACTTCAGGCTCTACTACCTCCAGCAGCTCCCTGCTGAGCCTGGAAAAGGAGGAAAAAGTAAAGAATAAGCCAGGGGAAATCAGACACAACAGAGCCCCAACTAGGTTTCATGGGTAGCATAGGGAAGTGGTTAAAAAACTAAAAGGATAGATCCATTAATGAGGTAACAAATTATTGCCTTCATGTTGGGACAGAACAGGGCCAAATGGAAAAGAATGAAAGAGAAAGACAGATAGACACACACACACACACACACACACACAACACACACACACACACACACAGAGAGAGAGAGAACGAGCTCAGTGAATTGTCCAGGTGACACACTGATGAGGGAGTAACAGGACACTCTGAGTTAGTGCCCTCAGGACACACAGCATACAGGGATCATGAAAAGACTGTGCTCAATAATTTTCCATAAAATGTGCTCAAGTTTCCATGCAGTCGCCATGAGAATACAGTTTTTGAAGTCTGGTCCACCTACAGTAGGTTAGTAAATGATAAGGGGAGGAAGAAATGGAAACCTAAATATCTACTGCAATGAAAACCAACAGCAATGTTAGTAGGAATAATTCAGGCTTGCTTGAAAAGATGTAATCGATAATGTCAGCCCGCTCTGTTTTCCCTGAACCAGGAGTCTCCAGATGTCAACACAGAAGTAGCTGTTCACAATTGCTCAGTTACCTGGGGCATGGTGGGCCTTGGTCTTCTTCCTCTTCTTGGTCCTTTTTAATTCCTGCAATACATTCAGACAGGGACAGACAAAATAAGCCAATTCACCTACACCCATAACAGTCCACTGTCTAATCCCCACACAGGGATCTCAGGCTCCTCAGCATGAGAACAGGACAATGTGAGAGATATACTTCAGGAGG

human m2 NBPF consensus

CCTGAAAGCTGGTCATGATATTCTTTGGTTTGCATCTCAGAACCAAGGGTGAAATATCCCCATTCTGGTAGATCGTTATCCCAAAATCATTTATCCCAAGTTTGTGCAAACAGTTATGCCTTATTGTTCCCATCAGTTCAAAGAAAATGCCCCAGATGATTTCTAGGAGGAAAACTGCAGTATTCAGCCCTGTCTCATCAAATGCCCAGCTCGTTCATGGATGCAAGAATTTTAGACACTGAAATTAGAATGAAGGAGGAAATCTACAAACCCTTGAGTCCAAATCATACTTCTGTGAATTTTTTACATCTGCCTGGGTCCAATGTGCTGAGAGCGGGCTCAGGTTGCCACAGGCATGGCTGGAGACTAGGAATAGAGCCTTGCTCACTGACCCATTTCATGTCTAGGCTTCCAACTGAGACTACAGTTTCATTACAACCTATATGCGCCCATAGGTCCTGCCTGCGGCAATGACATCTCTCGGGTCAGTAAGGGCCACTTGGAACAGGAATATCACCCCTATCTGGAAGACCAGGTGGAGGCTTATCACCTTCACAGTAAGGTACTCACTGTCCACGTCAAGAGCCAAGCCAAGGTACTGTTCCTCCAATGAGTAAACAGCACTGCTGTAGGGCTGGCCTAAGTCAGGCAGTTCAAGATAACCTGAAGGAGTCGAATAACATCTATCCAGTGAGTCCTGCAAGACTTCAGGCTCTTTCTCATCCAGCAGCTCCCTGCTGAGCCTGGAAAAGTAGGAAAAAGTAAAGAATAAGCCAGGGGGAATCAGAAACCACACAGCCCCAGCTAGATTTCATGGCTAACATAAGGAACTGTTTAAAAAGAAAAAGGACAGATCCATTAATGAGGTAATGAATTATTGCCTTTATGTTGGGATAGACCAGGGCCAGGTAGAAAAGAATGAAAGAGAAAGACAGGGAGAGGGAGGAGAGAGAGAGAGAGGAGAAAGTGAGCTCAGCGAATTGGCCGGGTGACACACTGATGAAGGGGTCAAAGGACACTCTGAGTTAGTGCCCTCGGGACACACAGCGAACAGTGATCATGAAAAGAGTGGGCTCAATAATTTTCCATAAACTTGCTCAAGATTCCATGCAGTTGCCATACAGCCTTTGAGGTATGGTCAACCTATAGTAAGTTAGTAAATGATAAGGGGAGGAAGAAATGGAAACCTAAACATCTACTGCAATGAAAACCAACAGCAATGTCAGTAGGAGTAATTCAACCTTCGTTGAAAACATGAAATTGAACACACTCTTGTTTTCCCTGGACCTGGCATCTCCAGGTGTCAACACAGAATTAAGCATCCATAATTGCTCAAAGTTACCTGGGGCATGATGGGTCTTGGTCTTCTTCCACTTCTTGGTACTTTTCAATTTCTGCAATAAGTTCAGACATGGACAGACATATTAAGCTGGTTCTCCTACACACATAACAATCCACTGTCTAATCCTCACACAGGGACTTCAGGCTCCTCAGCATGAGAATAGGACACTGTGAGAGATAGTCTTCAGGAGGCCTGAAGG

human m3 NBPF consensus

CCTGAAGGCTGATCACCATAGAGATTCCTTGGTTTTTGTCCCAGAAACTGTGGGTAAAATTCCCTATTCTGGTAGATCGTTATCCCAATATCATTTGTCCCAAGTTTGTGCAAATGGTTATGCCATATTTTTCCAATCGATTTAAAGCAAATGCCCCCAAATGGTTGCTAGGAGAAAAACTGCACTATTCAGCCCTGTCTCATCAAATACTCAGATTGTTCATGGTAGCGAGGATTTTAGACGCTGAAATTAGAGTGAAGGATGAAATCTACAAGATCTACAAAATTGAGACAAAATCAGAGTTGTGTGAATTTGTCACATCTGCCCAGGTCCAACGTCATGAGAGTAGGATTAGGGCGCCACAGGCATGGCCTGAGACTAGGAAGAGAGCCTTGCTCACTGACCCATCCCTTGTCTGGGCTTCCAAGTGGAACTAGAGTTTCATTCAACCTACATGTGCCTATAGGTCCTCCCTGTGGCAATGACATCTCTCAGCTCAGTAAGGGCCACTTGCAGTAGGAATATGACCCTAACCAGAAGACTCAGTGGATCCTTATCACCTTCATAGAAAGGTACTCACCATCCATGTCAACAGCCAAGCCAACACGCTGTTGCTCCAATACGTAAAAGGCACTTCTGTAGGGCTGGCATGAGTCAGTCAGTTCAAGACAACCTGAAGGAGTTGAATAACATCTATCCAGTGAGTCCTGCAAGACTTCAGGCCCTTTCTCATCCAGCAGCTCCCTGCTGAGCCTGGAAAAGTGGGAAAAAGTAAAGAATAAGCCAGGGGGAATCAGAAACCACACAGCCCCAGCTAGATTTCATGGCTAACGTAAGGAAGAGTTTGAAAAGAAAAAGGACAGATCCATTAATGAGGTAACAAATTATTGCCTTTATGTTGGGATAGAACAGGGCCAGGTAGAAAACAATGAAAGAGAAAGACAGACAGAGAGAGACAAAGAAGAGACAGAGACAGAGACAGAGAGAAAGTGACCTAGTGAATTGGCCAGGTGACATACTGGTAAGGGAGTCAAAGGACACTCTGAGTTAGTGCCCTCATGACACACAGCAAACTGTGATCATGAAAAGAGTGAGCTCAATAGTTTTCCATAAAATATGCTCAAAATTCGATGCAGTGGCCATGAGAGTACAGCTTTTGAAGTATGGTCAACCTATGGTACGTTAGGAAATGATAAGGGGAGGAAGAAATGGAAACCTAAACATCTACTGCAATGAAAACCAACAGCAATGACAGTAGGAGTAATTCAGCCTTCGCTGAAAACATGACATCAAACACACTCTGGTTTCCCTGAATCTGTTGCCTCCAGGTGTTAACACAGAATTAAGCATCCACAATTGCTGAAAGTCACCTGGGGCATGGTGGGTTTTGATCTTCTTCCCCTTCTTTTCTTCCCCTTCTTCTTTCCTTCTTTGATCTTCTTCCCCTTCTTTTCTTCCCCTTCCCCTTCTTTTCAATTTCTGCAATAAATTCAGACATGGACAGACACATTAAGCTGATTCCCCTACACACATAACAATCCACTGTCTAATCCTCACACAGGGACCTCAGGCTCCTCAGCATAAGAATAGGACACTGTGAGAGATATATTTCAGGAGGCCTGAGCGT
