# Supplementary material for: Tandemly repeated NBPF HOR copies (Olduvai triplets): Possible impact on human brain evolution
Source: Life Sci Alliance. 2022 Oct 19;6(1):e202101306. doi: 10.26508/lsa.202101306 (PMC9584774; doi:10.26508/lsa.202101306)
Supplement: Supplementary file 2 [file LSA-2021-01306_TableS2.docx]

**Supplementary Table 2.** Distribution of NBPF monomers and 3mer HORs in primate genomes (human, chimpanzee, gorilla, orangutan, rhesus macaque).

**Human**

| **Position** | **Momomer** | **Orientation** | **Divergence (%)** |
| --- | --- | --- | --- |
| 16565065 | m2 | plus | 9.47 |
| 16566598 | m3 | plus | 15.86 |
| 21425628 | m1 | plus | 10.98 |
| 21479230 | m2 | minus | 17.46 |
| 21480786 | m1 | minus | 11.30 |
| 120460379 | m3 | minus | 14.75 |
| 120461995 | m2 | minus | 1.10 |
| 120463536 | m1 | minus | 1.26 |
| 120823098 | m3 | minus | 15.86 |
| 120824718 | m2 | minus | 9.80 |
| 120826179 | m3 | minus | 13.83 |
| 120829616 | m2 | minus | 0.84 |
| 120831151 | m1 | minus | 1.39 |
| 120832728 | m3 | minus | 1.29 |
| 120836369 | m2 | minus | 0.71 |
| 120837908 | m1 | minus | 1.01 |
| 143596196 | m1 | plus | 15.72 |
| 143597726 | m1 | plus | 15.47 |
| 144424115 | m1 | plus | 2.27 |
| 144425704 | m2 | plus | 1.23 |
| 144427230 | m3 | plus | 15.55 |
| 145292627 | m1 | plus | 0.88 |
| 145294211 | m2 | plus | 2.08 |
| 145295757 | m3 | plus | 1.84 |
| 145297386 | m1 | plus | 1.39 |
| 145298977 | m2 | plus | 1.49 |
| 145300519 | m3 | plus | 1.78 |
| 145302139 | m1 | plus | 1.45 |
| 145303720 | m2 | plus | 7.85 |
| 145305317 | m1 | plus | 1.01 |
| 145306903 | m2 | plus | 7.85 |
| 145308500 | m1 | plus | 0.88 |
| 145310087 | m2 | plus | 1.30 |
| 145311627 | m3 | plus | 1.97 |
| 145313256 | m1 | plus | 2.21 |
| 145314855 | m2 | plus | 1.69 |
| 145316397 | m3 | plus | 2.03 |
| 145318028 | m1 | plus | 2.02 |
| 145319625 | m2 | plus | 0.45 |
| 145321159 | m3 | plus | 2.03 |
| 145322790 | m1 | plus | 0.69 |
| 145324375 | m2 | plus | 0.45 |
| 145325909 | m3 | plus | 1.84 |
| 145327538 | m1 | plus | 2.02 |
| 145329135 | m2 | plus | 1.56 |
| 145330676 | m3 | plus | 1.78 |
| 145332304 | m1 | plus | 0.76 |
| 145333888 | m2 | plus | 1.95 |
| 145335432 | m3 | plus | 1.78 |
| 145337060 | m1 | plus | 0.44 |
| 145338644 | m2 | plus | 1.56 |
| 145340186 | m3 | plus | 1.29 |
| 145341811 | m1 | plus | 0.95 |
| 145343398 | m2 | plus | 1.56 |
| 145344940 | m3 | plus | 1.84 |
| 145346569 | m1 | plus | 2.27 |
| 145348168 | m2 | plus | 1.62 |
| 145349710 | m3 | plus | 2.09 |
| 145351341 | m1 | plus | 1.45 |
| 145352932 | m2 | plus | 1.62 |
| 145354474 | m3 | plus | 1.72 |
| 145356096 | m1 | plus | 1.33 |
| 145357680 | m2 | plus | 1.49 |
| 145359221 | m3 | plus | 1.78 |
| 145360849 | m1 | plus | 0.76 |
| 145362433 | m2 | plus | 1.49 |
| 145363975 | m3 | plus | 1.84 |
| 145365604 | m1 | plus | 1.20 |
| 145367193 | m2 | plus | 1.62 |
| 145368735 | m3 | plus | 1.78 |
| 145370357 | m1 | plus | 1.26 |
| 145371941 | m2 | plus | 1.69 |
| 145373483 | m3 | plus | 1.78 |
| 145375111 | m1 | plus | 0.69 |
| 145376695 | m2 | plus | 0.97 |
| 145378233 | m3 | plus | 2.09 |
| 145379863 | m1 | plus | 0.82 |
| 145381447 | m2 | plus | 1.56 |
| 145382989 | m3 | plus | 1.78 |
| 145384618 | m1 | plus | 0.69 |
| 145386203 | m2 | plus | 0.45 |
| 145387737 | m3 | plus | 2.09 |
| 145389368 | m1 | plus | 1.33 |
| 145390959 | m2 | plus | 0.45 |
| 145392492 | m3 | plus | 15.30 |
| 145575066 | m1 | plus | 3.16 |
| 145576650 | m2 | plus | 3.50 |
| 145578202 | m3 | plus | 15.92 |
| 146067427 | m2 | plus | 5.65 |
| 146068957 | m3 | plus | 1.97 |
| 146070571 | m1 | plus | 0.63 |
| 146072157 | m2 | plus | 0.78 |
| 146073679 | m3 | plus | 2.83 |
| 146075293 | m1 | plus | 2.71 |
| 146076893 | m2 | plus | 1.04 |
| 146078415 | m3 | plus | 2.77 |
| 146080029 | m1 | plus | 1.83 |
| 146081623 | m2 | plus | 1.04 |
| 146083151 | m3 | plus | 2.03 |
| 146084765 | m1 | plus | 1.20 |
| 146086350 | m2 | plus | 1.30 |
| 146087872 | m3 | plus | 2.77 |
| 146089486 | m1 | plus | 0.57 |
| 146091068 | m2 | plus | 1.23 |
| 146092590 | m3 | plus | 2.70 |
| 146094204 | m1 | plus | 0.95 |
| 146095790 | m2 | plus | 1.17 |
| 146097312 | m3 | plus | 2.77 |
| 146098926 | m1 | plus | 0.95 |
| 146100512 | m2 | plus | 1.17 |
| 146102034 | m3 | plus | 2.64 |
| 146103648 | m1 | plus | 0.44 |
| 146105230 | m2 | plus | 1.23 |
| 146106752 | m3 | plus | 2.77 |
| 146108357 | m1 | plus | 1.58 |
| 146109940 | m2 | plus | 1.30 |
| 146111462 | m3 | plus | 2.40 |
| 146113075 | m1 | plus | 0.76 |
| 146114658 | m2 | plus | 1.23 |
| 146116180 | m3 | plus | 2.40 |
| 146117794 | m1 | plus | 1.45 |
| 146119384 | m2 | plus | 1.43 |
| 146120916 | m3 | plus | 1.29 |
| 146122530 | m1 | plus | 0.95 |
| 146124117 | m2 | plus | 1.56 |
| 146125649 | m3 | plus | 16.04 |
| 146983938 | m3 | minus | 15.73 |
| 146985553 | m2 | minus | 2.14 |
| 146987110 | m1 | minus | 4.99 |
| 146988696 | m3 | minus | 3.07 |
| 146990314 | m2 | minus | 2.14 |
| 146991871 | m1 | minus | 4.23 |
| 147112874 | m2 | plus | 15.31 |
| 148104784 | m1 | plus | 2.97 |
| 148106368 | m2 | plus | 1.95 |
| 148107901 | m3 | plus | 15.67 |
| 148534108 | m2 | plus | 5.84 |
| 148535643 | m3 | plus | 1.11 |
| 148537255 | m1 | plus | 0.57 |
| 148538836 | m2 | plus | 1.17 |
| 148540364 | m3 | plus | 1.91 |
| 148541969 | m1 | plus | 1.58 |
| 148543550 | m2 | plus | 1.10 |
| 148545078 | m3 | plus | 1.91 |
| 148546677 | m1 | plus | 2.08 |
| 148548258 | m2 | plus | 1.17 |
| 148549786 | m3 | plus | 1.84 |
| 148551385 | m1 | plus | 2.15 |
| 148552966 | m2 | plus | 1.17 |
| 148554499 | m3 | plus | 1.41 |
| 148556097 | m1 | plus | 2.78 |
| 148557680 | m2 | plus | 1.17 |
| 148559213 | m3 | plus | 0.98 |
| 148560831 | m1 | plus | 2.15 |
| 148562425 | m2 | plus | 0.97 |
| 148563953 | m3 | plus | 1.84 |
| 148565558 | m1 | plus | 2.08 |
| 148567141 | m2 | plus | 1.04 |
| 148568674 | m3 | plus | 1.48 |
| 148570288 | m1 | plus | 0.88 |
| 148571862 | m3 | plus | 9.34 |
| 148573477 | m1 | plus | 1.39 |
| 148575068 | m2 | plus | 0.97 |
| 148576601 | m3 | plus | 15.06 |
| 149056759 | m1 | plus | 1.64 |
| 149058354 | m2 | plus | 9.93 |
| 149059942 | m1 | plus | 2.27 |
| 149061519 | m2 | plus | 1.43 |
| 149063052 | m3 | plus | 14.75 |
| 149487138 | m3 | minus | 15.12 |
| 149488757 | m2 | minus | 1.36 |
| 149490302 | m1 | minus | 1.33 |
| 149491881 | m3 | minus | 2.64 |
| 149493512 | m2 | minus | 1.69 |
| 149495060 | m1 | minus | 1.52 |
| 149496644 | m3 | minus | 2.03 |
| 149498269 | m2 | minus | 1.49 |
| 149499815 | m1 | minus | 1.45 |
| 149501399 | m3 | minus | 2.64 |
| 149503030 | m2 | minus | 1.49 |
| 149504576 | m1 | minus | 1.45 |
| 149506160 | m3 | minus | 2.64 |
| 149507791 | m2 | minus | 1.49 |
| 149509337 | m1 | minus | 1.45 |
| 149510921 | m3 | minus | 2.64 |
| 149512552 | m2 | minus | 1.36 |
| 149514098 | m1 | minus | 0.88 |
| 149515677 | m3 | minus | 2.83 |
| 149517308 | m2 | minus | 1.04 |
| 149518851 | m1 | minus | 0.88 |
| 149520430 | m3 | minus | 2.95 |
| 149522063 | m2 | minus | 0.97 |
| 149523607 | m1 | minus | 1.33 |
| 149525187 | m3 | minus | 2.09 |
| 149526814 | m2 | minus | 0.78 |
| 149528337 | m1 | minus | 3.03 |
| 149529916 | m3 | minus | 2.21 |
| 149531543 | m2 | minus | 0.97 |
| 149533087 | m1 | minus | 0.82 |
| 149534667 | m3 | minus | 2.83 |
| 149536298 | m2 | minus | 0.97 |
| 149537839 | m1 | minus | 1.52 |
| 149539418 | m3 | minus | 2.40 |
| 149541045 | m2 | minus | 1.95 |
| 149542576 | m1 | minus | 3.66 |
| 149544155 | m3 | minus | 2.27 |
| 149545782 | m2 | minus | 2.01 |
| 149547313 | m1 | minus | 3.60 |
| 149548892 | m3 | minus | 2.27 |
| 149550519 | m2 | minus | 1.95 |
| 149552050 | m1 | minus | 3.60 |

**Chimpanzee**

| **Position** | **Momomer** | **Orientation** | **Divergence (%)** |
| --- | --- | --- | --- |
| 4089079 | m3 | plus | 1.23 |
| 19701271 | m1 | plus | 2.46 |
| 19702851 | m2 | plus | 1.56 |
| 19704385 | m3 | plus | 2.03 |
| 19778361 | m1 | plus | 4.99 |
| 19779920 | m2 | plus | 1.10 |
| 20043463 | m3 | minus | 13.15 |
| 20045090 | m1 | minus | 11.24 |
| 112181716 | m1 | plus | 3.98 |
| 112183281 | m2 | plus | 1.36 |
| 112184794 | m3 | plus | 3.20 |
| 120580500 | m1 | plus | 3.98 |
| 120582065 | m2 | plus | 1.04 |
| 121526197 | m2 | minus | 15.12 |
| 121638721 | m1 | plus | 3.41 |
| 121640290 | m2 | plus | 1.30 |
| 121641820 | m3 | plus | 1.48 |
| 122356823 | m3 | plus | 9.34 |
| 123165213 | m3 | minus | 3.32 |
| 123166832 | m2 | minus | 1.49 |
| 123168373 | m1 | minus | 3.35 |
| 124221428 | m2 | plus | 15.06 |
| 124222957 | m3 | plus | 17.76 |
| 124224509 | m2 | plus | 17.91 |
|  |  |  |  |

**Gorilla**

| **Position** | **Momomer** | **Orientation** | **Divergence (%)** |
| --- | --- | --- | --- |
| 17212139 | m1 | plus | 12.12 |
| 17213741 | m2 | plus | 16.16 |
| 17333393 | m2 | minus | 17.65 |
| 17334939 | m1 | minus | 11.87 |
| 115988199 | m1 | plus | 2.15 |
| 116359940 | m3 | minus | 14.75 |
| 116361557 | m2 | minus | 1.04 |
| 116363080 | m1 | minus | 3.41 |
| 116864584 | m3 | minus | 15.43 |
| 116866202 | m2 | minus | 1.17 |
| 116867727 | m1 | minus | 3.28 |
| 118065675 | m2 | minus | 15.38 |
| 118192230 | m1 | plus | 6.44 |
| 118193832 | m2 | plus | 14.67 |
| 120122765 | m2 | plus | 14.99 |
| 120124262 | m1 | plus | 14.02 |

**Orangutan**

| **Position** | **Momomer** | **Orientation** | **Divergence (%)** |
| --- | --- | --- | --- |
| 101306286 | m1 | minus | 14.84 |
| 103851927 | m1 | minus | 12.75 |
| 104101839 | m3 | minus | 16.53 |
| 104103455 | m2 | minus | 4.09 |
| 104104973 | m1 | minus | 7.70 |
| 105072254 | m2 | minus | 15.70 |
| 105166801 | m1 | plus | 8.52 |
| 105168399 | m2 | plus | 14.02 |
| 106111064 | m2 | minus | 16.29 |
| 106112608 | m1 | minus | 7.26 |
| 196507511 | m1 | minus | 10.54 |
| 196509107 | m2 | minus | 16.81 |
| 211269869 | m1 | plus | 11.30 |
| 211271469 | m2 | plus | 17.72 |

**Rhesus macaque**

| **Position** | **Momomer** | **Orientation** | **Divergence (%)** |
| --- | --- | --- | --- |
| 103011554.00 | m2 | minus | 13.37 |
| 103825672.00 | m2 | minus | 16.42 |
| 103827228.00 | m1 | minus | 11.68 |
| 133041578.00 | m2 | minus | 16.74 |
| 133043082.00 | m1 | minus | 11.99 |
| 208097919.00 | m1 | minus | 13.76 |
