# Supplementary material for: Tandemly repeated NBPF HOR copies (Olduvai triplets): Possible impact on human brain evolution
Source: Life Sci Alliance. 2022 Oct 19;6(1):e202101306. doi: 10.26508/lsa.202101306 (PMC9584774; doi:10.26508/lsa.202101306)
Supplement: Supplementary file 3 [file LSA-2021-01306_TableS3.docx]

**Supplementary Table 3.** Twenty randomly chosen individual human genomes (10 male, 10 female) from The International Genome Sample Resource (IGSR) (The 1000 Genomes Project: <https://www.internationalgenome.org/data-portal>).

| **Sample** | **Seks** | **Populations** | **Biosample ID** | **Cell line source** |
| --- | --- | --- | --- | --- |
| HG00107 | male | British in England and Scotland, European Ancestry | SAME123947 | HG00107 at Coriell |
| HG00121 | female | British in England and Scotland, European Ancestry | SAME122873 | HG00121 at Coriell |
| HG00359 | female | Finnish in Finland, European Ancestry | SAME125127 | HG00359 at Coriell |
| HG00366 | male | Finnish in Finland, European Ancestry | SAME124541 | HG00366 at Coriell |
| HG00525 | female | Han Chinese South, East Asian Ancestry | SAME123242 | HG00525 at Coriell |
| HG00532 | male | Han Chinese South, East Asian Ancestry | SAME125268 | HG00532 at Coriell |
| HG00640 | male | Puerto Rican in Puerto Rico, American Ancestry | SAME123434 | HG00640 at Coriell |
| HG01162 | female | Puerto Rican in Puerto Rico, American Ancestry | SAME1839707 | HG01162 at Coriell |
| HG01345 | female | Colombian in Medellin, Colombia, American Ancestry | SAME124863 | HG01345 at Coriell |
| HG01491 | male | Colombian in Medellin, Colombia, American Ancestry | SAME124140 | HG01491 at Coriell |
| HG01509 | male | Iberian populations in Spain, European Ancestry | SAME124940 | HG01509 at Coriell |
| HG01605 | female | Iberian populations in Spain, European Ancestry | SAME123459 | HG01605 at Coriell |
| HG01794 | female | Chinese Dai in Xishuangbanna, China, East Asian Ancestry | SAME124218 | HG01794 at Coriell |
| HG00866 | male | Chinese Dai in Xishuangbanna, China, East Asian Ancestry | SAME123549 | HG00866 at Coriell |
| HG01597 | female | Kinh in Ho Chi Minh City, Vietnam, East Asian Ancestry | SAME125226 | HG01597 at Coriell |
| HG01864 | male | Kinh in Ho Chi Minh City, Vietnam, East Asian Ancestry | SAME123197 | HG01864 at Coriell |
| HG01888 | male | African Caribbean in Barbados, African Ancestry | SAME122852 | HG01888 at Coriell |
| HG01915 | female | African Caribbean in Barbados, African Ancestry | SAME1839757 | HG01915 at Coriell |
| HG01922 | female | Peruvian in Lima, Peru, American Ancestry | SAME123787 | HG01922 at Coriell |
| HG01934 | male | Peruvian in Lima, Peru, American Ancestry | SAME123589 | HG01934 at Coriell |
