# Supplementary material for: Tandemly repeated NBPF HOR copies (Olduvai triplets): Possible impact on human brain evolution
Source: Life Sci Alliance. 2022 Oct 19;6(1):e202101306. doi: 10.26508/lsa.202101306 (PMC9584774; doi:10.26508/lsa.202101306)
Supplement: Supplementary file 4 [file LSA-2021-01306_TableS4.docx]

**Supplementary Table 4.** Large ( > 1 kbp) unsequenced gaps in chimpanzee NCBI referent genome assembly NC_036879.1.

| Positio | | Length (bp) |
| --- | --- | --- |
| start | end |  |
| 11.509.071 | 11.516.576 | 7.505 |
| 19.941.934 | 19.974.289 | 32.355 |
| 23.848.377 | 23.929.707 | 81.330 |
| 70.812.876 | 70.998.789 | 185.913 |
| 82.023.747 | 82.211.433 | 187.686 |
| 82.294.026 | 82.416.870 | 122.844 |
| 108.185.908 | 108.294.823 | 108.915 |
| 111.861.442 | 111.954.570 | 93.128 |
| 112.401.096 | 112.568.090 | 166.994 |
| 112.721.577 | 113.342.907 | 621.330 |
| 116.466.791 | 116.495.019 | 28.228 |
| 119.819.313 | 120.120.942 | 301.629 |
| 121.685.064 | 121.883.423 | 198.359 |
| 122.360.815 | 122.562.488 | 201.673 |
| 122.710.087 | 122.826.473 | 116.386 |
| 122.940.370 | 122.977.392 | 37.022 |
| 134.440.684 | 134.690.240 | 249.556 |
| 136.418.572 | 136.521.484 | 102.912 |
| 149.698.815 | 149.753.333 | 54.518 |
| 171.924.441 | 171.961.078 | 36.637 |
| 172.096.401 | 172.097.416 | 1.015 |
| 203.582.007 | 203.593.435 | 11.428 |
| 217.768.293 | 217.816.312 | 48.019 |
| 223.741.069 | 223.745.773 | 4.704 |
